# Significance of Wild-Type p53 Signaling in Suppressing Apoptosis in Response to Chemical Genotoxic Agents: Impact on Chemotherapy Outcome

**DOI:** 10.3390/ijms18050928

**Published:** 2017-04-28

**Authors:** Razmik Mirzayans, Bonnie Andrais, Piyush Kumar, David Murray

**Affiliations:** Department of Oncology, University of Alberta, Cross Cancer Institute, Edmonton, AB T6G 1Z2, Canada; bonnie.andrais@ahs.ca (B.A.); pkumar@ualberta.ca (P.K.); david.murray5@ahs.ca (D.M.)

**Keywords:** chemical genotoxic agents, p53 signaling, p21^WAF1^ (CDKN1A), DNAJB9, multinucleated giant cells, premature senescence, apoptosis, mutational processes

## Abstract

Our genomes are subject to potentially deleterious alterations resulting from endogenous sources (e.g., cellular metabolism, routine errors in DNA replication and recombination), exogenous sources (e.g., radiation, chemical agents), and medical diagnostic and treatment applications. Genome integrity and cellular homeostasis are maintained through an intricate network of pathways that serve to recognize the DNA damage, activate cell cycle checkpoints and facilitate DNA repair, or eliminate highly injured cells from the proliferating population. The wild-type p53 tumor suppressor and its downstream effector p21^WAF1^ (p21) are key regulators of these responses. Although extensively studied for its ability to control cell cycle progression, p21 has emerged as a multifunctional protein capable of downregulating p53, suppressing apoptosis, and orchestrating prolonged growth arrest through stress-induced premature senescence. Studies with solid tumors and solid tumor-derived cell lines have revealed that such growth-arrested cancer cells remain viable, secrete growth-promoting factors, and can give rise to progeny with stem-cell-like properties. This article provides an overview of the mechanisms by which p53 signaling suppresses apoptosis following genotoxic stress, facilitating repair of genomic injury under physiological conditions but having the potential to promote tumor regrowth in response to cancer chemotherapy.

## 1. Introduction

Our cells are continuously exposed to potentially deleterious genotoxic events from both endogenous and exogenous sources that jeopardize genome integrity. The plethora of DNA lesions include DNA strand breaks and base alterations induced by ionizing radiation and chemical agents that generate reactive oxygen species, DNA alkylation and formation of abasic sites induced by alkylating agents, bulky DNA lesions induced by ultraviolet light (UV), DNA interstrand crosslinks induced by bifunctional alkylating agents and platinum drugs, and DNA-protein crosslinks arising from a wide range of chemicals, such as chemotherapeutic drugs and formaldehyde [1,2,3,4]. Constitutively available DNA repair processes deal with low levels of genomic injury and assist in ameliorating the detrimental effects of such agents. An increase in DNA damage above a threshold level activates the DNA damage surveillance network, which involves multiple signaling pathways that protect against genomic instability and restrict aberrant cell growth in response to genotoxic stress [5]. The wild-type p53 tumor suppressor functions at the hub of this network [6,7].

In the mid 1990s it was proposed that the principal role of p53 in determining cell fate following genotoxic stress is to either promote survival by activating cell cycle checkpoints and facilitating DNA repair or induce apoptotic cell death. This two-armed model of the DNA damage surveillance network—namely, repair and survive, or die through apoptosis—provided the impetus for extensive research directed towards modulating p53 in an attempt to improve the outcome of conventional cancer therapies. However, it soon became clear that p53’s function extends beyond canonical cell cycle and apoptotic signaling, and impacts additional diverse biological processes including senescence and metabolism [8,9]. Murine cancer models have been employed to investigate the impact of p53 activation in the response of oncogene-driven cancers. As pointed out by Stegh [9], “*confirming important roles of p53 in cancer suppression, these studies showed that reactivation of p53 in established tumors can temporarily stop tumor growth; the precise cellular mechanism is cancer type-specific, as lymphomas die by apoptosis, whereas p53 restoration in sarcomas and liver carcinomas leads to growth arrest and senescence. p53-driven apoptosis and senescence responses associated with temporary p53 reactivation led to prolonged survival. Although cancer remission was not permanent, and p53-resistant tumors emerged…*” The promises, challenges and perils of targeting p53 in cancer therapy have been extensively discussed [8,9,10].

A growing body of evidence suggests that the primary response triggered by moderate, clinically relevant doses of cancer therapeutic agents is a sustained proliferation block and not apoptosis in most human cell types (e.g., dermal fibroblasts, solid tumor-derived cells) [6,11], with activation of p53 signaling suppressing (rather than promoting) apoptosis [12,13,14]. Such growth-arrested cells remain viable for long times (months) post-treatment, secrete a myriad of biologically active factors, and can give rise to progeny exhibiting stem-cell-like properties.

Herein we briefly review the mechanisms by which wild-type p53 suppresses apoptosis following genotoxic stress, focusing on the roles played by DNAJ homolog subfamily B member 9 (DNAJB9) and p21^WAF1^ (p21; also called CDKN1A). In addition, we discuss the significance of p53-mediated protection against apoptosis under physiological conditions, and the dark side of this function of p53 in the context of cancer chemotherapy.

## 2. Biological Outputs Orchestrated by Wild-Type p53

### 2.1. p53 Functions

Wild-type p53 is a multifunctional tumor suppressor capable of activating transient cell cycle checkpoints, accelerating DNA repair processes including nucleotide excision repair and rejoining of DNA double strand-breaks (DSBs), and eliminating highly injured cells from the proliferating population by inducing stress-induced premature senescence (SIPS) or apoptotic cell death [6]. p53 exerts these effects both directly, through protein–protein interaction (e.g., interacting with key mediators of DNA repair and apoptosis [6,15]), and indirectly by transcriptionally activating p21 and other key players in the DNA damage surveillance network [6,16].

SIPS is a sustained growth arrested state resembling replicative senescence, a hallmark of mammalian cell aging [17]. Both events are characterized by the acquisition of flattened and enlarged cell morphology and expression of the marker senescence associated β-galactosidase (SA-β-gal) in cells that retain viability and exhibit metabolic activity. Unlike replicative senescence, which is triggered by erosion and dysfunction of telomeres, SIPS is induced by DNA damage and other types of genotoxic stress but is not dependent on telomere status and telomerase function [17]. Both events are largely (but not always) dependent on wild-type p53 signaling in general, and sustained nuclear accumulation of p21 in particular.

SIPS, triggered by DNA-damaging agents, is a prominent response of normal human fibroblasts and solid tumor-derived cell lines that express wild-type p53 [6]. In addition, Li–Fraumeni syndrome fibroblasts [18] and some lung carcinoma cell lines [19] that lack wild-type p53 function also exhibit a high degree of SIPS in response to genotoxic stress (ionizing radiation). SIPS in p53-deficient cells correlated with induction of p16^INK4A^ (p16) but not of p21, leading us to propose that p16 might function in a redundant pathway of senescence (both replicative senescence and SIPS), triggering this process only in the absence of wild-type p53 activity [18]. Interestingly, p16 has been reported to be repressed in a p53-dependent manner. Hernández-Vargas et al. [20], for example, reported that p53 transcriptionally activates the helix-loop-helix transcriptional regulator protein Id1, a well-known repressor of *p16^INK^*^4^*^A^* [21,22]. In addition, Leong et al. [23] demonstrated that p53 downregulates p16 through Id1-independent mechanisms.

### 2.2. p53 Regulation in the Absence of Genotoxic Stress

In normal, unstressed cells, the wild-type p53 protein undergoes rapid turnover and is thus maintained at low steady state levels that restrict its function [6,7]. Turnover of p53 is controlled by several ubiquitin ligases, some of which are regulated in a p53-dependent manner. MDM2 (murine double minute-2 homologue; also known as HDM2 in human) is the most intensively studied regulator of p53 stability and function. In the absence of DNA damage, MDM2 binds to the N-terminal region of p53 and inhibits its activity by blocking p53-mediated transactivation, exporting p53 from the nucleus to the cytoplasm, and promoting the proteasomal degradation of p53. MDM2-mediated mono-ubiquitination of p53 triggers its cytoplasmic sequestration, whereas poly-ubiquitination results in p53 degradation.

### 2.3. p53 Regulation Following Genotoxic Stress

Recent studies have revealed that a threshold level of genotoxic stress must be reached to trigger the DNA damage surveillance network [5]. This response is initiated by rapid stabilization of p53, its nuclear accumulation, and activation of its transcriptional and biological functions [24]. Stabilization and activation of p53 is largely a consequence of phosphorylation of the molecule on different residues, which can be mediated by various protein kinases, including ATM (ataxia telangiectasia mutated), ATR (ATM and RAD3-related), checkpoint kinase 1 (CHK1), checkpoint kinase 2 (CHK2), and p38 mitogen-activated protein kinase (MAPK) [25,26,27,28]. In response to DNA damage, phosphorylation of p53 on Ser20 and of MDM2 on Ser395, mediated by kinases such as ATM, interrupts the p53–MDM2 interaction, resulting in p53 accumulation, subcellular shuttling and activation [7].

Rapid activation of the DNA damage surveillance network in response to genotoxic stress must be followed by restoration of the cell to its pre-stress state to allow the maintenance of cell homeostasis and resumption of normal growth. This critical function is largely accomplished by WIP1 (wild-type p53-induced phosphatase 1), a p53-regulated type 2C serine/threonine phosphatase [29].

### 2.4. p53 Dynamics Following Genotoxic Stress

The mechanism by which a single tumor suppressor, p53, orchestrates complex responses to DNA damage has been the subject of extensive research. Much attention has been focused on the function of p53 and its downstream programs at relatively short times (within hours) after genotoxic insult. In 2004, Lahav and associates [30] reported studies with the MCF7 breast carcinoma cell line demonstrating that the temporal dynamics of p53 following DNA damage constitutes another potential level of regulation for different biological outcomes. Immunoblot and single-cell observation methods revealed that p53 levels rise and fall in a wavelike or “pulsed” manner in response to DNA double-strand breaks induced by ionizing radiation. Both MDM2 [30] and WIP1 [31] were shown to contribute to the negative regulation of p53 at various p53 waves. These observations led the authors to propose a model in which the initial p53 waves would allow the cells to activate cell cycle checkpoints to facilitate repair, and the subsequent waves to determine cell fate.

These ground-breaking discoveries provided an impetus for a number of studies involving mathematical simulations that were designed to uncover the basis for the “digital” p53 response and the biological consequences of different p53 waves. As discussed previously [6,32], most such studies assumed that the ultimate cell fate might reflect apoptosis, even in MCF7 cells which are relatively insensitive to undergoing apoptosis consequent to therapeutic exposures [33,34,35]. Purvis et al. [36], however, determined the predominant cell fate resulting from p53 dynamics post-irradiation and showed this to be SIPS in MCF7 cells. We have reported a similar outcome with the A172 malignant glioma cell line [32].

Advances and perspectives regarding the dynamics and mathematical models of p53 signaling in response to different types of DNA damage, together with insight into the biological functions of such dynamics, have been extensively reviewed [32,37] and will not be considered further.

### 2.5. A Threshold Mechanism Determines the Choice Between p53-Mediated Growth Arrest versus Apoptosis

The biological output of p53 signaling in response to genotoxic stress in terms of sustained growth arrest or apoptotic cell death depends on several factors, including the amount and type of genotoxic insult and the genetic background of the cells [38,39,40]. As extensively discussed recently [6,32], in most human cell types (e.g., non-cancerous skin fibroblast strains and solid tumor-derived cell lines), exposure to moderate doses of genotoxic agents (e.g., ionizing radiation, UV, chemotherapeutic drugs) promotes a high degree of SIPS but only marginal (if any) apoptosis. Moderate doses refer to those that are typically used in the in vitro colony formation assay and are relevant to in vivo therapeutic studies with animal models. Exposure to extremely high doses of such agents, resulting in <1% clonogenic survival, triggers apoptosis in a significant proportion (~50%) of the cells. (The importance of the apoptotic threshold for exposure to cancer chemotherapeutic agents will be considered in Section 5.1.)

Recently, Kracikova et al. [41] determined the influence of p53 expression levels on biological outcomes in the absence of genotoxic stress. These authors used two approaches to achieve conditions where the only variable is the level of p53: (i) an inducible system with human epithelial cells that allows tight regulation of p53 expression; and (ii) human cancer cells treated with the p53 activator nutlin-3. Both approaches demonstrated that low and high p53 expression triggered growth arrest and apoptosis, respectively. Consistent with these observations, real-time PCR, microarray and ChIP analyses showed that p53 binds to and transcriptionally activates both pro-arrest and pro-apoptotic target genes proportionally to its expression levels. However, low levels of p53 pro-arrest proteins initiated the growth-arrested response, whereas low levels of pro-apoptotic proteins failed to trigger apoptosis. The authors concluded that their observations “*suggest a mechanism whereby the biological outcome of p53 activation is determined by different cellular thresholds for arrest and apoptosis. Lowering the apoptotic threshold was sufficient to switch the p53 cell fate from arrest to apoptosis, which has important implications for the effectiveness of p53-based cancer therapy*.” Growth arrest in these experiments was judged from accumulation of cells in the G0/G1 phase of the cell cycle [41]. In other studies, nutlin-3-triggered activation of p53 signaling was shown to result in marginal apoptosis but a high degree of growth arrest through SIPS in p53 wild-type cancer cells [42,43,44,45].

### 2.6. Anti-Apoptotic Property of p53 Signaling under Physiological Conditions

**Figure 1:**
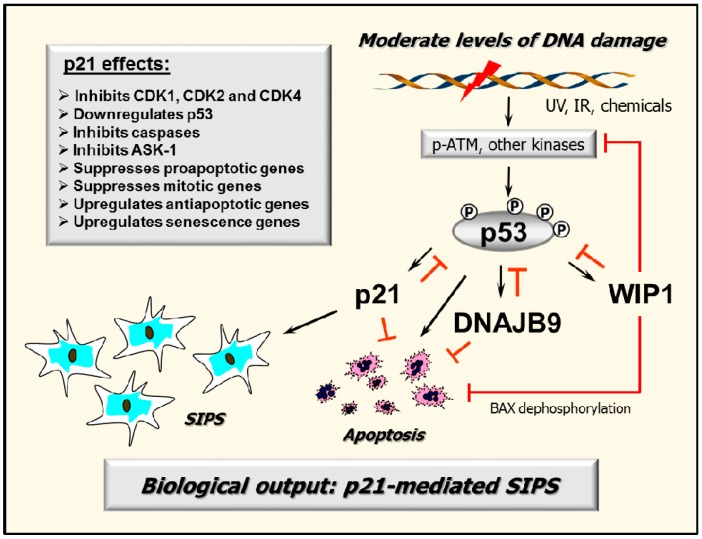
A partial schematic of the DNA damage surveillance network illustrating the importance of negative regulation of p53 by p21, DNAJ homolog subfamily B member 9 (DNAJB9), and wild-type p53-induced phosphatase 1 (WIP1) in suppressing apoptosis as discussed in this article. Arrows indicate stimulation and T-shaped lines indicate inhibition. Multiple functions of p21 in the DNA damage surveillance network are indicated.

Under some conditions, p53 is known to activate apoptotic signaling (but not necessarily cell death) both directly, through its proline-rich region, and indirectly by inducing the expression of pro-apoptotic proteins such a BAX (BCL-2-associated X protein), PUMA (p53 upregulated modulator of apoptosis) and NOXA (the Latin word for damage) [6,46]. Simultaneously, under the same conditions, p53 also transcriptionally activates a host of anti-apoptotic proteins, including p21, 14-3-3δ, WIP1 and DNAJB9 [6,46,47]. Thus, in most cell types (e.g., cells derived from solid tumors), activation of p53 signaling not only fails to promote apoptotic cell death (i.e., cell demise), it actually protects against this response. As extensively discussed by Jänicke et al. [46], the anti-apoptotic and transient growth inhibitory properties of p53 “*are surely essential for normal development and maintenance of a healthy organism, but may easily turn into the dark side of the tumor suppressor p53 contributing to tumorigenesis.*” In their article, which was published in 2008 [46], these authors considered approximately 40 p53-regulated proteins that exhibit anti-apoptotic properties. Below we will limit our discussion to DNAJB9 and p21, both of which participate in a negative regulatory loop with p53 (Figure 1).

### 2.7. p53–DNAJB9 Regulatory Loop: Impact on Apoptosis

DNAJB9 (DNAJ homolog subfamily B member 9) functions in many cellular processes by regulating the ATPase activities of the 70 kDa heat shock proteins (Hsp70s). Recently, Lee et al. [47] identified DNAJB9 as a transcriptional target of p53 in human cancer cell lines. Employing Western and Northern blot analyses, p53-dependent expression of DNAJB9 was demonstrated in both overexpression studies with EJ-p53, a human bladder carcinoma cell line that expresses p53 under the control of a tetracycline-regulated promoter, and with p53 wild-type cancer cell lines (e.g., SKNSH neuroblastoma) after treatment with doxorubicin and other chemotherapeutic agents. Immunofluorescence experiments demonstrated that DNAJB9 co-localizes with p53 in both the cytoplasm and nucleus after genotoxic stress. DNAJB9 depletion and overexpression studies demonstrated that this p53-regulated protein inhibits the pro-apoptotic function of p53 through a physical interaction. Thus, DNABJ9 suppresses apoptosis in response to chemotherapeutic agents by forming a negative regulatory loop with p53.

### 2.8. Multiple Functions of p21: Downregulating p53 and More

The p21 protein was discovered by different groups in the early 1990s and was variously called WAF1 (for wild-type p53-activated fragment 1), CIP1 (for CDK-interacting protein 1), and SDI1 (for senescent cell-derived inhibitor 1) [48,49]. It has been extensively studied for its ability to influence cell cycle progression by inhibiting the activity of cyclin/cyclin dependent kinase (CDK) complexes (e.g., CDK1, 2 and 4). In 2002, Javelaud and Besançon [50] reported an additional function for p21 in the DNA damage surveillance network. Disruption of p21 expression in HCT116 colorectal carcinoma cells, either by gene targeting or gene silencing by using antisense oligonucleotides, resulted in an increase in p53 steady-state levels in the absence of genotoxic treatment. Elevated expression of p53 in p21-depleted HCT116 cells correlated with high expression of p14^ARF^, the product of an alternative transcript of the *INK4A* locus, which is known to promote p53 stability through binding to its negative regulator, MDM2 [51,52]. In addition, elevated expression of p53 in p21-depleted cells resulted in marked sensitivity to chemotherapeutic drug-induced cytotoxicity through activation of the mitochondrial pathway of apoptosis. Thus, p21 may indirectly participate in the regulation of p53 protein stability through preventing p14^ARF^-mediated MDM2 breakdown, resulting in marked resistance towards stress-induced apoptosis.

Since then, numerous reports have established the broad-acting functions of p21 beyond its influence on the cell cycle. For example, we recently demonstrated that one mechanism by which p21 exerts its inhibitory effects on p53 and apoptosis is through regulating WIP1, an oncogenic phosphatase that inactivates p53 and its upstream kinases [53]. Other groups have demonstrated that the anti-apoptotic property of p21 also relies on its ability to inhibit the activity of proteins directly involved in the induction of apoptosis, including the caspase cascade, stress-activated protein kinases (SAPKs) and apoptosis signal-regulating kinase 1 (ASK1) [54,55,56], and to control transcription, resulting in downregulation of pro-apoptotic genes [56] and upregulation of genes that encode secreted factors with anti-apoptotic activities [55,56].

It is noteworthy that in a review article published in 2012 [32], we suggested that p21 might function as a positive regulator of p53 in the DNA damage surveillance network. This notion was based on a report suggesting that loss of p21 in the HCT116 cell line led to cytoplasmic sequestration of p53 and inhibition of its transcriptional activity [57]. This observation, however, was not confirmed by us [53] and others [58]. On the contrary, we found that loss of p21 in this cell line results in robust accumulation of p53, and that p53 molecules are phosphorylated (e.g., on Ser15) and accumulated in the nucleus even in the absence of exogenous stress [53]. Accordingly, we and others have concluded that p21 downregulates p53, at least in the HCT116 colon carcinoma [50,53,58,59], MCF7 breast carcinoma [53], and HT1080 fibrosarcoma [59] cell lines.

In addition to its strong anti-apoptotic properties, p21 also plays a key role in orchestrating the complex SIPS program in cells expressing wild-type p53 [6,60]. Studies with cancer cell lines treated with chemotherapeutic agents demonstrated that p21 forms a positive regulatory loop with ATM and that this interaction is essential for the maintenance of the growth-arrested response, a hallmark of SIPS [32,61]; pharmacological targeting of either p21 or ATM triggers apoptosis of growth-arrested cancer cells [61].

Some authors use the term “arrest” without clearly distinguishing between transient G1/S checkpoint activation and SIPS. As discussed recently [6], these two responses are uncoupled, at least in human skin fibroblast strains and solid tumor-derived cell lines. In these cell types, G1/S checkpoint activation following exposure to DNA-damaging agents is an early event required to provide time for the repair of genomic injury before resumption of the cell cycle, whereas SIPS is manifested at late times (several days) post-treatment. Multiple factors contribute to the regulation of SIPS, including p21-mediated expression of a battery of genes involved in growth arrest, senescence, and aging, coupled with p21-mediated downregulation of numerous genes that control mitosis [17,55].

To summarize, the pivotal role of p21 in determining cell fate in response to genotoxic stress is not only through activating the G1/S cell cycle checkpoint, but also through controlling gene expression, suppressing apoptosis by acting at different levels of the death cascade, and promoting growth arrest through SIPS.

## 3. Activation of Apoptotic Signaling Does Not Always Lead to Cell Death: Impact on Chemosensitivity Assessment

It is now widely accepted that transcriptional activation of pro-apoptotic proteins (e.g., PUMA, NOXA, BAX) might not inevitably lead to cell death as a result of concomitant activation of a host of anti-apoptotic proteins that maintain p53 under the apoptotic threshold level (e.g., MDM2, p21, WIP1, DNAJB9) [6,47], sequester pro-apoptotic factors such as BAX (e.g., 14-3-3δ) [62,63,64,65,66], and inhibit ASK1 and the caspase cascade (e.g., p21) [6]. Similarly, while caspase 3 functions as a key apoptosis executioner under some conditions, such as in the development and maintenance of the hematopoietic system, under other conditions it reveals its dark side by promoting tumor growth [67,68,69,70,71,72,73,74,75,76,77]. For these and several other reasons, the Nomenclature Committee on Cell Death (NCCD) has cautioned the scientific community about the use/misuse of terminologies and concepts in the area of cell death research. Notably, in their 2009 article, the NCCD pointed out that bona fide “dead cells” would be different from “dying cells” that have not crossed the point of no return and have not concluded their demise [78]. It is worth noting that radiosensitivity and chemosensitivity, assessed by the widely-used multi-well plate colorimetric assays, which determine the inhibition of cell growth (resulting from the combined impact of checkpoint activation, growth inhibition and cytotoxicity), have often been misinterpreted to reflect loss of viability and hence cell death.

**Figure 2:**
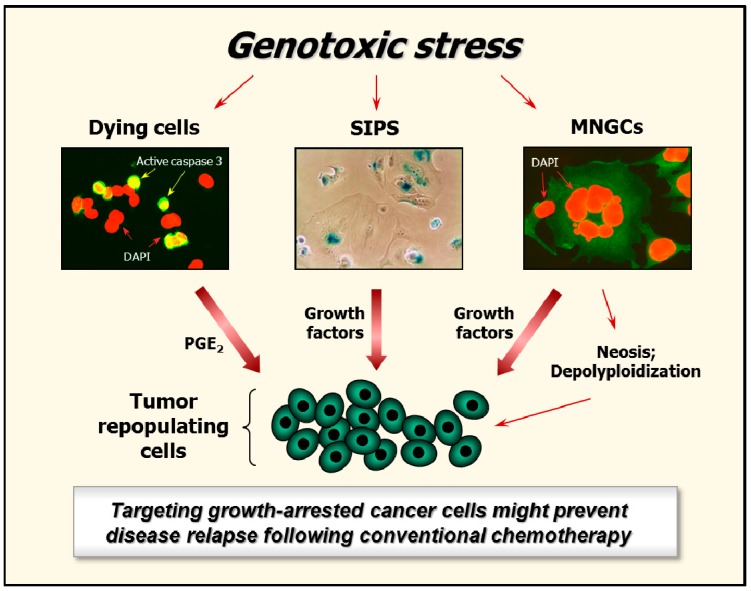
Examples of genotoxic stress-induced responses associated with cancer cell death or survival depending on context: Activation of caspase 3, induction of stress-induced premature senescence (SIPS), and creation of multinucleated giant cells (MNGCs). SIPS is a genetically-controlled process, mediated by p21 or p16, depending on the p53 status of the cells [6,11]. MNGCs can be created through different routes, including endoreduplication (replication of chromosomes without subsequent cell division) and homotypic cell fusions [11].

Recently we reviewed the current knowledge on responses induced by ionizing radiation that can lead to cancer cell death or survival depending on the context [11]. These include activation of caspases (e.g., caspase 3), growth arrest through SIPS, and creation of polyploid/multinucleated giant cells (hereafter called MNGCs) (also see Figure 2). Such potentially pro-survival responses are triggered not only by ionizing radiation, but also by chemotherapeutic drugs [74,79,80,81,82,83,84] and hypoxia [72,85,86,87,88,89].

Caspase 3 is extensively studied for its role in the execution phase of apoptosis [90]. Accordingly, the activated (cleaved) form of caspase 3 has often been used as a molecular marker of apoptosis. Paradoxically, in recent years caspase 3 has also been demonstrated to function as a survival factor, promoting the growth of tumor-repopulating cells [68,69,70,71,72,73,74,75,76,77]. This pro-survival effect of caspase 3 has been attributed to secretion of prostaglandin E_2_ (PGE_2_) [70,72,74]. The caspase 3-PGE_2_ survival pathway is triggered by various stimuli, including ionizing radiation [70,74], chemotherapeutic drugs [72,74] and hypoxia [72]. Interestingly, the biological outcome associated with caspase 3 activation is in part dependent on p21 (reviewed in [32]). Thus, p21-mediated inhibition of caspase 3 activity results in suppression of apoptosis in response to genotoxic stress. On the other hand, caspase 3-mediated cleavage of p21 generates a 15 kDa fragment of p21 that appears to positively regulate apoptosis by forming a complex with active caspase 3. It is currently unknown whether p21 might play a role in the regulation of the caspase 3-PGE_2_ survival pathway.

Whether caspase 3 plays a role in growth-arrested cancer cells also remains to be elucidated. However, it is well known that cancer cells undergoing SIPS remain viable and acquire the ability to secrete factors that can promote proliferation and invasiveness in cell culture models and tumor development in vivo [91,92]. This so-called “senescence-associated secretory phenotype” (SASP) includes several families of soluble and insoluble factors that can affect surrounding cells by activating various cell surface receptors and corresponding signal transduction pathways [91,92].

While some authors consider SASP to be the “dark” side of senescence [91,92,93,94,95,96], others have proposed that induction of senescence (SIPS) might be advantageous for cancer treatment [97,98,99,100,101]. As pointed out by Maier et al. [101], although “*accumulation of senescent cancer cells leads to an increased secretion of inflammatory cytokines, which might cause age-related pathologies, like secondary cancers, in the long term, the primary aim of cancer treatment leading to a longer overall survival should always take preference. Thus, the hypothetical possibility that senescent cells may be dormant with an intrinsic capability to reawaken years after the treatment is of secondary concern, similar to the risk of inducing second cancers.*” However, aside from SASP, there is now compelling evidence that cancer cells undergoing SIPS can themselves give rise to stem-cell-like progeny, thereby contributing to cancer relapse following therapy [11,102,103].

Like cells undergoing SIPS, MNGCs also remain viable and secrete cell-growth promoting factors [11]. This property of MNGCs was first reported over 60 years ago for HeLa cervical carcinoma cells exposed to ionizing radiation [104,105,106]. HeLa cells harbor wild-type alleles of *TP53*, but are infected with human papillomavirus (HPV) 18, the E6 protein of which disables the p53–p21 axis [107]. This observation prompted Puck and Marcus to develop the feeder layer clonogenic assay, in which a “lawn” of heavily-irradiated feeder cells (which encompass MNGCs) is inoculated into a culture dish to promote the growth of test cells given graded doses of genotoxic agents [105]. Recently, we demonstrated that exposure of a panel of p53-deficient or p21-deficient solid tumor-derived cell lines to moderate doses of ionizing radiation (e.g., 8 Gy) results in the development of MNGCs that remain adherent to the culture dish, retain viability, metabolize 3-(4,5-dimethylthiazol-2-yl)-2,5-diphenyl-tetrazolium bromide (MTT), and exhibit DNA synthesis for long times (e.g., three weeks) post-irradiation [108].

Collectively, these observations underscore the importance of distinguishing between dead cells and growth arrested cells that might be mistakenly scored as “dead” in the colony formation and other cell-based radiosensitivity/chemosensitivity assays. As pointed out recently [108], the creation of viable growth arrested cells (e.g., MNGCs) complicates the interpretation of data obtained with multi-well plate colorimetric tests routinely used in anti-cancer drug-screening endeavors.

## 4. Extrapolating Results Obtained in Overexpression Studies to Clinically Relevant Conditions

The preceding discussion raises a fundamental question with respect to p53 regulation and function. As discussed by Uversky [7], p53 undergoes extensive post-translational modifications (e.g., phosphorylation, acetylation) that are critical for its stabilization and activation. Such modifications result in accumulation of p53 in the nucleus and the formation of p53 tetramers, which then bind to the promoters of target genes and trigger their expression. Genotoxic stress activates factors such as ATM and ATR that initiate the DNA damage surveillance network by mediating p53 posttranslational modifications (also see Figure 1). Despite the wealth of knowledge regarding the importance of genotoxic stress (e.g., DNA damage) in activating the p53-mediated transcriptional program, this same transcriptional program has also been reported to be activated by ectopic expression of wild-type p53 without exposure to exogenous stress [41]. Does this indicate that stress-triggered p53 posttranslational modifications are not needed for activation of its transcriptional program, which appears to be highly unlikely, or does p53 overexpression by itself create a non-physiological condition that it is sufficient to trigger the stress response?

It is important to note that many reports suggesting a positive role for wild-type p53 in triggering apoptosis, either with or without exposure to genotoxic agents, involved overexpression experiments with a variety of transformed/malignant cell types (e.g., T-cell leukemia cell lines). In addition, many authors did not follow the NCCD recommendations to distinguish between “dying cells” (i.e., exhibiting transient activation of a death-related biochemical pathway) and cells that are irreversibly committed to die. Taken together with the observations of Kracikova et al. [41] described above (Section 2.5.), demonstrating that different expression levels of exogenous p53 yield different outcomes (G1 arrest versus apoptotic signaling), caution should be exercised in extrapolating results obtained in overexpression studies to clinically relevant conditions (e.g., cancer chemotherapy) particularly when it pertains to p53-directed cancer cell death.

## 5. Fate of Growth-Arrested Cancer Cells

### 5.1. Clinically Relevant Doses of Chemotherapeutic Agents Predominantly Trigger Cancer Cell Dormancy Rather Than Cell Death

Studies with some cell types (e.g., solid tumor-derived cell lines) have shown that many (if not all) chemotherapeutic agents predominantly trigger growth arrest but not cell death when administered at clinically achievable concentrations [11]. Below, we mainly focus on cisplatin and solid tumors.

Berndtsson et al. [79] examined numerous articles, published between 2002 and 2005, which reported apoptosis after cisplatin treatment in a wide range of cell lines. The mean cisplatin concentration used to induce apoptosis was 52 μM; when examined, concentrations below 20 μM did not induce apoptosis but triggered growth arrest [79]. These and more recent studies have reported IC_50_ values (50% inhibiting concentrations) of >40 μM and <2 μM for induction of apoptosis and growth arrest by cisplatin, respectively [11]. We have observed a similar trend with a panel of cancer cell lines expressing wild-type p53 (HCT116, A549, MCF7), mutant p53 (MDA-MD-231, SUM159) or no p53 (HCT116 p53 knockout). In all cell lines, a 3-day incubation with 10 μM cisplatin resulted in growth inhibition of all cells (IC_50_ values ranging from 0.5 to 2 μM), but did not induce cytotoxicity when evaluated by the vital dye (trypan blue) exclusion and other assays (data not shown).

The finding that very high concentrations of cisplatin are required to induce apoptosis in solid tumor-derived cell lines is not surprising given that this effect has been reported to primarily reflect cisplatin-induced injury to mitochondria rather than to nuclear DNA [79]. This raises the important question as to whether high, apoptosis-triggering concentrations of cisplatin are relevant for in vivo studies and, by inference, for treating cancer patients. Puig et al. [82] have addressed this question using a rat colon carcinoma cell line grown both in vitro and in vivo. Treatment of animals with cisplatin concentrations corresponding to those which induced apoptosis in the cell-based (tissue culture) experiments caused major toxic side effects on the gastrointestinal tract, bone marrow and kidney. When administered at tolerated concentrations (corresponding to ≤10 μM in cell culture experiments), cisplatin induced tumor cell dormancy (through SIPS and multinucleation) but did not kill tumor cells.

### 5.2. Hypoxia and the Creation of MNGCs

Hypoxia is one of the most important pathological features of solid tumors, and represents a major obstacle in cancer therapy [109,110]. Hypoxia constitutes a physiological selective pressure promoting tumor aggressiveness, which is largely associated with the maintenance and formation of cancer stem cells, promoting their phenotype and tumorigenesis. Many of the cellular responses to hypoxia are controlled by the transcription factor hypoxia-inducible factor-1 (HIF-1), which is a heterodimer composed of α and β subunits. HIF-1α contains two oxygen dependent degradation domains. Under normoxic conditions these domains are continuously hydroxylated by prolyl hydroxylases, resulting in HIF-1α degradation. Hypoxic conditions result in stabilization of HIF-1α. Stabilized HIF-1α accumulates in the nucleus where it binds to HIF-1β subunit, forming a transcription factor capable of activating the expression of numerous target genes, including those involved in energy production, angiogenesis, and metabolic adaptation to hypoxia [109,111].

Cobalt chloride (CoCl_2_) is used as a hypoxia mimicking agent when administered under normoxic conditions. CoCl_2_ stabilizes HIF-1α by inhibiting prolyl hydroxylase enzymes [112,113,114]. Several reports have demonstrated that treatment of human cancer cells with CoCl_2_ induces the formation of MNGCs through endoreduplication and/or cell fusion. MNGCs exhibit resistance to genotoxic agents (e.g., doxorubicin [115]) and give rise to tumor repopulating progeny through splitting, budding, or burst-like mechanisms (see also Section 5.3). CoCl_2_-triggered creation of MNGCs and emergence of their proliferating progeny has been reported for ovarian [88,116], breast [87] and colon [83] carcinoma cells. Studies with cancer cell lines as well as tumor tissues from cancer patients have identified several factors that appear to promote the survival of MNGCs and control their fate. These include the cell cycle regulatory proteins cyclin E, S-phase kinase-associated protein 2 (SKP2), and stathmin [88], as well as the epithelial–mesenchymal transition (EMT)-related proteins E-cadherin, N-cadherin, and vimentin [87].

### 5.3. Genome Reduction and Neosis of MNGCs

The observation that MNGCs created in response to genotoxic stress (ionizing radiation) remain viable and secrete cell growth-promoting factors was reported over half a century ago [104,105,106]. This seminal discovery was largely overlooked and with time the induction of massive genetic anomalies seen in MNGCs was often assumed to be associated with cell death through mitotic catastrophe. In 2000, Erenpreisa et al. [117] and Illidge et al. [118] reported that MNGCs that develop in heavily irradiated p53-deficient human cell cultures undergo a complex breakdown and sub-nuclear reorganization, ultimately giving rise to rapidly propagating daughter cells. The authors proposed that the development of MNGCs might represent a unique mechanism of “repair” enabling p53-deficient cancer cells to maintain proliferative capacity despite experiencing extensive genomic instability [117,118]. These initial experiments involved a Burkitt’s lymphoma cell line. The creation of MNGCs capable of generating proliferating daughter cells has now been reported from different laboratories for various cell types in response to a wide range of genotoxic agents, including chemotherapeutic drugs. This so-called “endopolyploidy-stemness” route of cancer-cell survival consequent to therapeutic exposure has also been documented with short-term (2–3 weeks) cultures of primary human breast cancer specimens [119].

One mechanism of depolyploidization of MNGCs is through genome reduction division (reviewed in [120]). In this process, MNGCs first undergo a ploidy cycle, which is regulated by key mediators of mitosis (e.g., aurora B kinase), meiosis (e.g., MOS), and self-renewal (e.g., OCT4), ultimately giving rise to a para-diploid progeny, containing a near-diploid number of chromosomes, that exhibit mitotic propagation. In 2004, Sundaram et al. [121] reported an alternative mechanism of genome reduction in MNGCs. Computerized video time-lapse microscopy revealed that, although MNGCs may cease to divide, each giant cell might produce numerous (50 or more) small cells with low cytoplasmic content (karyoplasts) via the nuclear budding process of “neosis” that resembles the parasexual mode of somatic reduction division of simple organisms like fungi. The resultant budding daughter cells begin to divide by mitosis and transiently display stem cell properties, and subsequently experience a complex life cycle eventually leading to the development of highly metastatic and therapy resistant descendants. This parasexual mode of somatic reduction division of MNGCs (neosis) has been reported from different laboratories for human ovarian [88,116], breast [87] and colon carcinoma cell lines [83] as well as other biological systems [122,123,124,125,126,127,128].

### 5.4. Is SIPS Reversible?

The creation of MNGCs and their proliferating daughter cells appears to be a general feature of cells that lack wild-type p53 function. Under some conditions, p53-proficient cells that undergo SIPS can also follow the polyploidy stemness route [11]. As an example, doxorubicin treatment of HCT116 colon carcinoma cell cultures resulted in growth-arrested cells that were positive in the SA-β-gal assay, but with time these same cells became polyploid and generated growing progeny [129]. In the same study, MCF7 breast cancer cells also exhibited growth arrest coupled with SA-β-gal staining following doxorubicin treatment, but this response was not accompanied by polyploidy and generation of proliferating progeny [129]. Although the basis for reversibility of SIPS in some contexts is not known, it is interesting to note that HCT116 cells are caspase 3 proficient, whereas MCF7 cells do not express caspase 3.

## 6. Targeting Growth-Arrested Cancer Cells as a Potential Therapeutic Strategy

The importance of MNGCs in the failure of cancer therapy has been largely overlooked. In part, this may be because the creation of such cells has been considered to be rare and also multinucleation has often been assumed to reflect death through “mitotic catastrophe” or other mechanisms. As discussed in this article, when administered at clinically relevant doses, cancer chemotherapeutic agents trigger a high proportion of MNGCs in solid tumor-derived cell lines (especially those lacking wild-type p53 function) that remain viable and can give rise to tumor repopulating progeny. Shockingly, only a single multinucleated giant cancer cell has been shown to be sufficient to cause metastatic disease when grafted under the skin of an animal [115]. Cancer cells undergoing SIPS in response to chemotherapeutic agents can also escape from the growth-arrested state and give rise to tumor-repopulating progeny.

Accordingly, targeting growth-arrested cancer cells might represent an effective therapeutic strategy. To this end, Crescenzi et al. [61] reported that downregulating either ATM or p21 in cancer cells that have undergone SIPS in response to chemotherapeutic drugs results in their demise. For targeting MNGCs for destruction, different approaches have been reported to be effective. These include viral infection [104] and treatment with pharmacological inhibitors of different members of the BCL-XL/BCL-2 pathway [130]. In addition, we have recently demonstrated that the apoptosis activators sodium salicylate (an inhibitor of the p38 MAPK) or dichloroacetate (a modulator of glucose metabolism) also kill MNGCs under conditions that have little or no effect on parental (mono-nucleated) cells [108]. The results of these proof-of-principle in vitro experiments are encouraging and warrant further studies with animal models.

## 7. Mutational Signatures in Human Cancers

The aforementioned reports concluding that the creation of MNGCs following chemotherapy might represent a survival mechanism for cancer cells involved studies not only with cultured cells and animal models, but also with patient specimens. Other studies, however, also reporting extensive experimental and clinical data, have concluded that this response might reflect a favorable therapeutic outcome (e.g., [131]). Similarly, the conclusions that SIPS might represent a favorable [97,98,99,100,101] or unfavorable [97,98,99,100,101,102,103] therapeutic outcomes have also been based on extensive experimental/clinical data. Such apparently conflicting observations might not be entirely unexpected when considering the distinct mutational types in aging and cancer.

**Figure 3:**
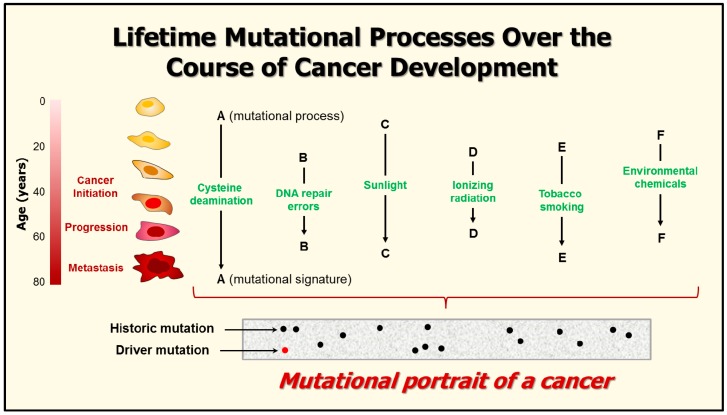
Cartoon showing mutational processes that can “scar” the genome during different periods of a person’s life span. The various mutations found in a tumor are grouped into “driver” mutations, which are ongoing and confer selective cancer phenotypes, and “historic” (or passenger) mutations which are far more numerous and hitchhike with driver mutations, but do not appear to be causative of cancer development. For details concerning ionizing radiation and other stimuli, consult [138] and [132,133,134,135], respectively. Adapted from Helleday et al. [135].

The advent of next-generation sequencing technologies has enabled large-scale sequencing of all protein-coding exons (whole-exome sequencing) or even whole cancer genomes (whole-genome sequencing) in a single experiment [132,133,134,135,136,137,138]. These sequencing efforts have enabled the identification of many thousands of mutations per cancer which provided sufficient power to detect different mutational patterns or “signatures.” Each biological perturbation or “mutational process” (e.g., tobacco smoke, sunlight exposure, deamination of DNA bases) is shown to leave a characteristic “mark” or mutational signature on the cancer genome (reviewed in [135]) (Figure 3).

Each mutational signature is defined by: (i) the type of genomic injury that has occurred as a result of a diversity of exogenous and endogenous genotoxic stresses; (ii) the integrity of DNA repair and other aspects of the DNA damage surveillance network that were successively activated; and (iii) the strength and duration of exposure to each mutational process. Additionally, as pointed out by Helleday et al. [135], “*cancers are likely to comprise different cell populations (that is, subclonal populations), which can be variably exposed to each mutational process; this promotes the complexity of the final landscape of somatic mutations in a cancer genome. The final “mutational portrait,” which is obtained after a cancer has been removed by surgery and then sequenced, is therefore a composite of multiple mutational signatures.*”

Thus, as previously anticipated, these large-scale sequencing technologies coupled with bioinformatic and computational tools for deciphering the “scars” (signatures) of mutational processes have demonstrated significant variability in the mutation landscape in cancers of the same histological type. Application of such approaches might similarly unfold the molecular basis for the fate of growth-arrested cancer cells in terms of death versus survival. This might in turn set the stage for designing novel therapeutic strategies for specifically targeting growth-arrested cancer cells before they will have the opportunity to generate tumor-repopulating progeny.

## 8. Conclusions

Inhibition of cell growth is an important response to genotoxic stress, either under physiological conditions or in cancer therapy. This response is fundamental for the maintenance of genomic stability and cellular homeostasis under physiological conditions. On the other hand, stress-induced growth arrest in cancer cells—reflecting either SIPS (predominantly in p53 wild-type cells) or the creation of MNGCs (predominantly in p53-deficient cells)—can provide a “survival” mechanism, ultimately resulting in the emergence of cancer repopulating progeny. Selective targeting of growth-arrested cancer cells (e.g., MNGCs) could represent a promising strategy for improving the outcome of conventional chemotherapy.

## Abbreviations

UV	Ultraviolet light
DSBs	DNA double-strand breaks
SA-β-gal	Senescence-associated β-galactosidase
SIPS	Stress-induced premature senescence
DNAJB9	DNAJ homolog subfamily B member 9
MDM2	Murine double minute-2 homologue
ATM	Ataxia telangiectasia mutated
ATR	ATM and RAD3-related
CHK	Checkpoint kinase
WIP1	Wild-type p53-induced phosphatase 1
BAX	BCL-2-associated X protein
PUMA	p53 upregulated modulator of apoptosis
WAF1	Wild-type p53-activated fragment 1
CIP1	CDK-interacting protein 1
SDI1	Senescent cell-derived inhibitor 1
CDK	Cyclin dependent kinase
SAPK	Stress-activated protein kinase
ASK1	Apoptosis signal-regulating kinase 1
HPV	Human papillomavirus
PGE_2_	Prostaglandin E_2_
SASP	Senescence-associated secretory phenotype
SKP2	*S*-phase kinase-associated protein 2
EMT	Epithelial-mesenchymal transition
MTT	3-(4,5-Dimethylthiazol-2-yl)-2,5-diphenyl-tetrazolium bromide
IC_50_	Inhibiting concentration, 50%
NCCD	Nomenclature Committee on Cell Death
